# Androgen Receptor Signaling Inhibitors for Metastatic Hormone Sensitive Prostate Cancer in Asians: Indirect Comparison

**DOI:** 10.1002/cnr2.70496

**Published:** 2026-02-16

**Authors:** Wei Chen, Soichiro Yoshida, Yasuhisa Fujii

**Affiliations:** ^1^ Department of Urology Institute of Science Tokyo Tokyo Japan; ^2^ Department of Urology Zigong Fourth People's Hospital Zigong Sichuan China

## Abstract

**Background:**

Androgen receptor signaling inhibitors (ARSI) combined with androgen deprivation therapy (ADT) have demonstrated significant survival benefits in metastatic hormone‐sensitive prostate cancer (mHSPC) in several clinical trials. However, data specifically in Asian patients remain limited, with individual subgroup analyses showing trends that did not reach statistical significance. We conducted a pooled analysis to provide additional evidence regarding the efficacy of ARSI plus ADT in Asian patients with mHSPC.

**Methods:**

We systematically identified clinical trials reporting overall survival (OS) outcomes for ARSI‐based doublet therapy in Asian patients with mHSPC. Individual patient data (IPD) were reconstructed from published Kaplan–Meier curves. Data sources included the TITAN Asian subpopulation analysis, the TITAN Japanese subpopulation, and the LATITUDE Japanese subpopulation. Reconstructed data were validated against original hazard ratios (HR) and curves. Pooled survival analyses were performed for the overall Asian cohort and separately for Japanese patients only.

**Results:**

A total of 291 reconstructed IPDs were analyzed (146 ARSI plus ADT, 145 placebo plus ADT). In the overall Asian cohort, the 3‐year OS was 85.2% with ARSI plus ADT versus 77.6% with placebo plus ADT, representing a 32% reduction in the risk of death (HR = 0.68, 95% CI 0.44–1.03, *p* = 0.07). In the Japanese subpopulation, 3‐year OS was 77.4% versus 72.4%, with a 45% reduction in death risk (HR = 0.55, 95% CI 0.29–1.04, *p* = 0.07). Neither analysis reached statistical significance.

**Conclusion:**

This exploratory analysis observed a trend toward improved survival with ARSI plus ADT in Asian patients with mHSPC that did not achieve statistical significance. Prospective studies are needed to validate these findings.

## 1 Introduction

Recent clinical trials have demonstrated the efficacy of androgen receptor signaling inhibitors (ARSI)‐based doublet or triplet therapy combinations in metastatic hormone‐sensitive prostate cancer (mHSPC) [1]. Our recent studies using reconstructed individual patient data (rIPD) analysis showed no significant improvement in overall survival (OS) in patients with mHSPC when docetaxel was added to ARSI‐based regimens [2, 3]. These results demonstrate the importance of combining ARSI with androgen deprivation therapy (ADT) in mHSPC management [4]. While randomized controlled trials, including TITAN [5] and LATITUDE [6] have shown significant advantages of ARSI plus ADT in the overall population, Asian patients often exhibit different characteristics compared to Western patients in terms of treatment response and prognosis [7]. The subgroup analyses of TITAN and LATITUDE have suggested potential benefits of ARSI plus ADT over ADT monotherapy for Asian patients, while the difference did not reach statistical differences [8, 9, 10]. We conducted a pooled analysis by reconstructing individual patient data (IPD) from these results and aim to provide further information about OS for the management of Asian patients.

## 2 Methods

### 2.1 Data Source and Ethics Statement

We systematically searched PubMed, Embase, and ClinicalTrials.gov through December 2025 to identify clinical trials evaluating ARSI‐based doublet therapy in Asian patients with mHSPC that reported Kaplan–Meier curves for OS. This study was conducted and reported in accordance with the PRISMA‐IPD guidelines for systematic reviews and meta‐analyses involving individual participant data. The analysis was based exclusively on published aggregate data and reconstructed time‐to‐event data derived from these sources; no identifiable individual‐level patient data were accessed. Accordingly, institutional review board approval and informed consent were not sought, in compliance with Japan's Ethical Guidelines for Medical and Biological Research Involving Human Subjects [11].

### 2.2 IPD Reconstruction

IPD were reconstructed from the published Kaplan–Meier curves using the IPDfromKM package in R software (version 4.3) as described by Liu et al. [12] Survival curves were digitized using Digitize software (FSF Inc., Boston, USA) to extract curve coordinates at approximately 128 to 448 time points per curve. Patient‐level time‐to‐event data were predicted by integrating the extracted coordinates with numbers at risk reported at specific time intervals in the original publications. The reconstruction algorithm estimated censoring times based on the reported numbers at risk and event occurrences at each time interval.

### 2.3 Risk of Bias Assessment

Given that this study was a secondary analysis based on rIPD from previously published randomized controlled trials, risk of bias was assessed at both the trial and reconstruction levels. At the trial level, methodological quality was evaluated based on published reports, including randomization, allocation procedures, completeness of follow‐up, and outcome ascertainment. All included studies were large phase III randomized controlled trials with predefined endpoints and standardized outcome definitions and were therefore considered to have a low overall risk of bias. At the reconstruction level, the rIPDs were validated through both qualitative and quantitative approaches. Qualitative validation included visual comparison by overlaying reconstructed curves with the original published curves. Quantitative validation incorporated the comparison of hazard ratios (HR) and 95% confidence intervals (CI) between reconstructed and original data.

### 2.4 Statistical Analysis

Survival analyses were performed using Cox proportional hazards models to calculate HR with 95% CI for OS, comparing ARSI plus ADT versus placebo plus ADT. Kaplan–Meier curves were generated for both treatment groups, and survival probabilities at 3 years were calculated. The log‐rank test was used to compare survival distributions between groups. All statistical analyses were conducted using R software version 4.3, with a two‐sided *p*‐value < 0.05 considered statistically significant.

## 3 Results

### 3.1 Basic Characteristics

A total of three reports have been screened out—TITAN analysis in the Japanese subpopulation [10], TITAN analysis in the Asian subpopulation [8], and the LATITUDE analysis in the Japanese subpopulation [9] (Table 1). The Asian population analysis from the TITAN included cohorts from Japan, China, and South Korea. To avoid duplicate counting of Japanese patients from TITAN (*n* = 51), data from the TITAN Asian subpopulation and LATITUDE Japanese subpopulation were pooled for the overall Asian cohort, resulting in a total of 291 reconstructed individual patient data points (146 ARSI plus ADT, 145 placebo plus ADT). Patient characteristics are summarized in Table 1. The median age ranged from 70 to 73 years across the studies. In TITAN, the majority of patients had ECOG performance status 0 (66.7%–89.3%), Gleason score > 7 (86.4%–96.4%), and approximately two‐thirds had high‐volume disease (64.3%–66.7%). LATITUDE exclusively enrolled high‐risk patients defined by ≥ 2 of the following: Gleason score ≥ 8, ≥ 3 bone lesions, or visceral metastases. The ARSI agents used were apalutamide 240 mg daily in TITAN and abiraterone 1000 mg daily in LATITUDE. The rIPDs demonstrated high concordance with the original published data, with HRs and 95% CIs closely matching the source reports (Table 2 and Figure S1–S3).

**TABLE 1:** Basic characteristics of included trials.

Characteristic	TITAN (Japanese subpopulation) [10]	TITAN (Asian subpopulation) [8]	LATITUDE (Japanese subpopulation) [9]
Design	Apalutamide + ADT (*n* = 28) vs. Placebo + ADT (*n* = 23)	Apalutamide + ADT (*n* = 111) vs. Placebo + ADT (*n* = 110)	Abiraterone + ADT (*n* = 35) vs. Placebo + ADT (*n* = 35)
Median age (range), years	73 (47–89) vs. 72 (62–84)	70 (47–89) vs. 70 (50–85)	Similar between groups
ECOG PS 0, *n* (%)	25 (89.3) vs. 19 (82.6)	74 (66.7) vs. 77 (70.0)	NA
Gleason score > 7, *n* (%)	27 (96.4) vs. 21 (91.3)	96 (86.5) vs. 95 (86.4)	NA
Metastatic stage at initial diagnosis (M1), *n* (%)	27 (96.4) vs. 21 (91.3)	104 (93.7) vs. 105 (95.5)	NA
High volume disease, *n* (%)	18 (64.3) vs. 15 (65.2)	74 (66.7) vs. 72 (65.5)	NA
Median PSA (range), ng/mL	NA	10.24 (0–2682) vs. 3.77 (0–803)	NA
Included Tumor Stage	mHSPC	mHSPC	mHSPC
ARSI drug and dosage	Apalutamide 240 mg qd.	Apalutamide 240 mg qd.	Abiraterone 1000 mg qd.
Metastatic stage	De novo & recurrent	De novo & recurrent	De novo only
Primary Endpoint	Overall survival	Overall survival	Overall survival, radiographic progression‐free survival
Risk criteria	All risk groups	All risk groups	High‐risk only (≥ 2 of: Gleason ≥ 8, ≥ 3 bone lesions, visceral metastases)

*Note:* Asian population includes patients from Japan, China, and South Korea. LATITUDE reported median age as similar between groups without specific values. Baseline characteristics for the LATITUDE Japanese subgroup were not fully reported in the original publication and are therefore unavailable.

Abbreviations: ADT, Androgen deprivation therapy; ARSI, androgen receptor signaling inhibitor; ECOG PS, The Eastern Cooperative Oncology Group Performance Status; mHSPC, Metastatic hormone‐sensitive prostate cancer; NA, Not reported; PSA, Prostate‐specific antigen.

**TABLE 2:** Quantitative validation of reconstructed data.

Trial	Original HR (95% CI)	Reconstructed HR (95% CI)	Z value	p value
LATITUDE (Japanese subpopulation)	0.61 (0.27–1.42)	0.61 (0.26–1.42)	0.00	1.00
TITAN (Japanese subpopulation)	0.45 (0.16–1.25)	0.47 (0.17–1.29)	−0.02	0.98
TITAN (Asian subpopulation)	0.69 (0.42–1.13)	0.70 (0.43–1.14)	−0.02	0.98

Abbreviations: CI, confidence interval; HR, hazard ratio.

### 3.2 Overall Cohort

**FIGURE 1:**
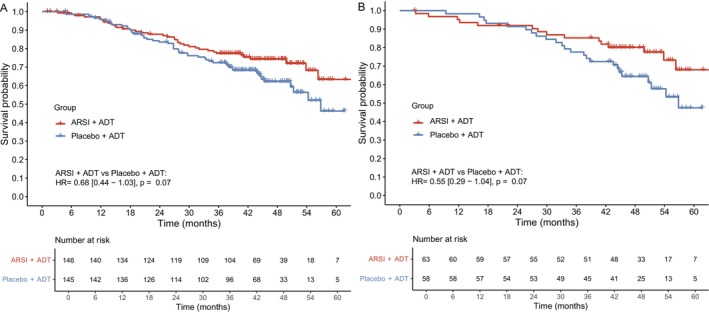
Overall survival in Asian patients with mHSPC treated with ARSI plus ADT versus placebo plus ADT. (A) Overall Asian cohort, showing overall survival comparing ARSI plus ADT versus placebo plus ADT. (B) Japanese subpopulation, showing overall survival in Japanese patients only. Survival curves were generated based on rIPD derived from published Kaplan–Meier curves. ADT, Androgen deprivation therapy; ARSI, androgen receptor signaling inhibitor; HR, Hazard ratio.

In the overall cohort, the 3‐year OS was 85.2% in the ARSI plus ADT group and 77.6% in the placebo plus ADT group. ARSI plus ADT provided a 32% reduction in the risk of death compared to placebo plus ADT (Figure 1A), with more pronounced differences after 2 years of follow‐up, though the differences did not reach statistical significance (HR = 0.68 [95% CI, 0.44–1.03], *p* = 0.07).

### 3.3 Japanese Subpopulation

Using the Japanese subpopulation from TITAN, the subgroup analysis was conducted for Japanese patients only from LATITUDE and TITAN. The 3‐year survival was 77.4% in the ARSI plus ADT group and 72.4% in the placebo plus ADT group. The result demonstrated that ARSI plus ADT improved OS by approximately 45% compared to placebo plus ADT, particularly after 2 years of follow‐up (Figure 1B). However, the same as the overall cohort, the difference also failed to reach statistical significance (HR = 0.55 [95% CI, 0.29–1.04], *p* = 0.07).

## 4 Discussion

Our pooled analysis observed a trend toward improved OS with ARSI plus ADT compared to placebo plus ADT in Asian patients with mHSPC; however, this trend did not achieve statistical significance. The observed hazard ratios of 0.68 in the overall Asian cohort and 0.55 in the Japanese subpopulation suggest potential benefit, but the lack of statistical significance precludes definitive conclusions regarding efficacy. While the point estimates align with the overall trial populations [8, 9, 10] (LATITUDE: HR = 0.66; TITAN: HR = 0.65), these findings must be interpreted with caution, given the exploratory nature of this analysis, which was not designed as a formal network meta‐analysis but rather as a pragmatic pooled analysis based on available subgroup‐level Kaplan–Meier data, intended for exploratory and hypothesis‐generating purposes.

Some limitations should be noted. First, the heterogeneity in patient backgrounds may introduce potential bias in comparison. Details of patient characteristics in LATITUDE are not reported. Based on the overall cohort, LATITUDE exclusively enrolled high‐risk mHSPC patients, defined as having at least two of three risk factors, whereas TITAN included patients across all risk categories. In the overall populations, LATITUDE patients had significantly higher proportions of high‐volume disease and Gleason score ≥ 8 compared to TITAN. These baseline differences may confound the pooled treatment effect estimate. Our inability to perform risk‐stratified analyses in the Asian cohort limits understanding of whether similar patterns exist in this population and whether the pooled hazard ratio accurately reflects benefit across different risk strata. Without IPD allowing for covariate adjustment, baseline imbalances between trials may introduce unpredictable bias. Furthermore, the ARCHES study evaluating enzalutamide was excluded as no published Asian subgroup analysis with OS data was available, limiting generalizability to all ARSI‐based regimens. In addition, the small sample size may contribute to potential bias in explaining the results. To date, only 3 studies derived from two trials are available for reconstructing the IPD in Asian cohorts. The lack of statistical significance may be due to the limited sample size. Nevertheless, the observed 32%–45% reduction in the risk of death aligns with the overall population. While the absence of individual patient‐level covariates prevents adjusted analyses that could account for confounding factors, further undermining the reliability of our findings. These results should be considered strictly exploratory and hypothesis‐generating, not as evidence to guide clinical practice. Furthermore, due to the limitation of original reports, we were unable to perform tumor volume‐specific or tumor risk‐specific analysis in the Asian population, although the difference may exist, particularly in patients with high‐volume or high‐risk disease. Finally, although we validated the rIPD in a precise way and reached consistency with the original curve, the rPD cannot fully reflect the original data. Our analyses were constrained by predefined comparisons and cannot adjust for covariates.

Despite these limitations, the consistency of treatment effect direction and magnitude with overall trial populations provides reassurance regarding the potential applicability of ARSI‐based therapy in Asian patients [13]. Prospective studies with adequate statistical power specifically designed for Asian populations are needed to definitively confirm these findings and identify optimal patient selection criteria.

In conclusion, this exploratory pooled analysis observed a trend toward improved overall survival with ARSI plus ADT in Asian patients with mHSPC, though statistical significance was not achieved. While the magnitude of benefit aligns with overall trial populations, prospective studies with adequate statistical power are needed to definitively establish the efficacy of ARSI‐based therapy in Asian populations.

In conclusion, this exploratory pooled analysis observed a trend toward improved overall survival with ARSI plus ADT in Asian patients with mHSPC, which may assist clinicians in treatment planning, particularly in aligning therapeutic expectations with the timing of clinical benefit. These findings can support patient counseling, help contextualize risk–benefit considerations during different phases of treatment, and facilitate more individualized application of existing trial evidence in routine clinical practice. While the magnitude of benefit aligns with overall trial populations, prospective studies with adequate statistical power are needed to definitively establish the efficacy of ARSI‐based therapy in Asian populations.

## Author Contributions

**Wei Chen:** writing – original draft, conceptualization, methodology, software, formal analysis, data Curation, visualization, funding acquisition; **Soichiro Yoshida:** writing – review and editing, conceptualization, methodology, data curation, supervision, project administration; **Yasuhisa Fujii:** writing – review and editing, supervision, project administration.

## Funding

This research was funded by the Health Commission of Sichuan Province Medical Science and Technology Program (24CXTD06) and the Sichuan Science and Technology Program (2026YFHZ0048).

## Ethics Statement

This study was conducted using reconstructed individual patient data derived exclusively from previously published Kaplan–Meier curves and aggregated summary statistics. No new patient data were collected, and no identifiable personal information was accessed or analyzed. This approach is consistent with the principles of the Declaration of Helsinki and the recommendations of the International Committee of Medical Journal Editors regarding secondary analyses of published data.

According to the Ethical Guidelines for Medical and Biological Research Involving Human Subjects in Japan, studies that exclusively use publicly available, anonymized data and do not involve direct interaction with human participants are exempt from institutional review board approval. Therefore, ethical approval and informed consent were waived.

## Consent

The authors have nothing to report.

## Conflicts of Interest

The authors declare no conflicts of interest.

## Supporting information


**Figure S1:** Validation of reconstructed individual patient data of overall survival in LATITUDE.
**Figure S2:** Validation of reconstructed individual patient data of overall survival in TITAN in Japanese subpopulation.
**Figure S3:** Validation of reconstructed individual patient data of overall survival in TITAN in Asian subpopulation.

## Data Availability

The data that support the findings of this study are available from the corresponding author upon reasonable request.
