# Supplementary material for: Androgen Receptor Signaling Inhibitors for Metastatic Hormone Sensitive Prostate Cancer in Asians: Indirect Comparison
Source: Cancer Rep (Hoboken). 2026 Feb 16;9(2):e70496. doi: 10.1002/cnr2.70496 (PMC12908864; doi:10.1002/cnr2.70496)
Supplement: Supplementary file 1 — Figure S1: Validation of reconstructed individual patient data of overall survival in LATITUDE. Figure S2: Validation of reconstructed individual patient data of overall survival in TITAN in Japanese subpopulation. Figure S3: Validation of reconstructed individual patient data of overall survival in TITAN in Asian subpopulation. [file CNR2-9-e70496-s001.docx]

**Supplementary files**

**
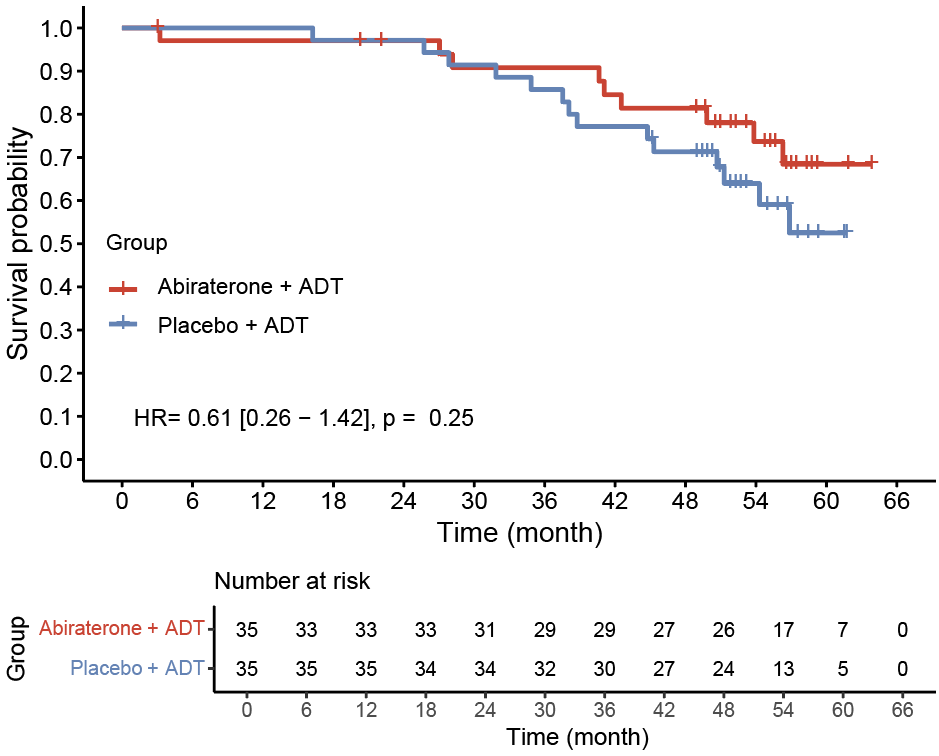
**

Figure s1. Reconstructed individual patient data (overall survival) in LATITUDE in Japanese subpopulation reconstructed from Figure 2a in Suzuki. Jpn J Clin Oncol. 2020, 50(7): 810-820.

KM, Kaplan-Meier; HR, hazard ratio; CI, confidence interval


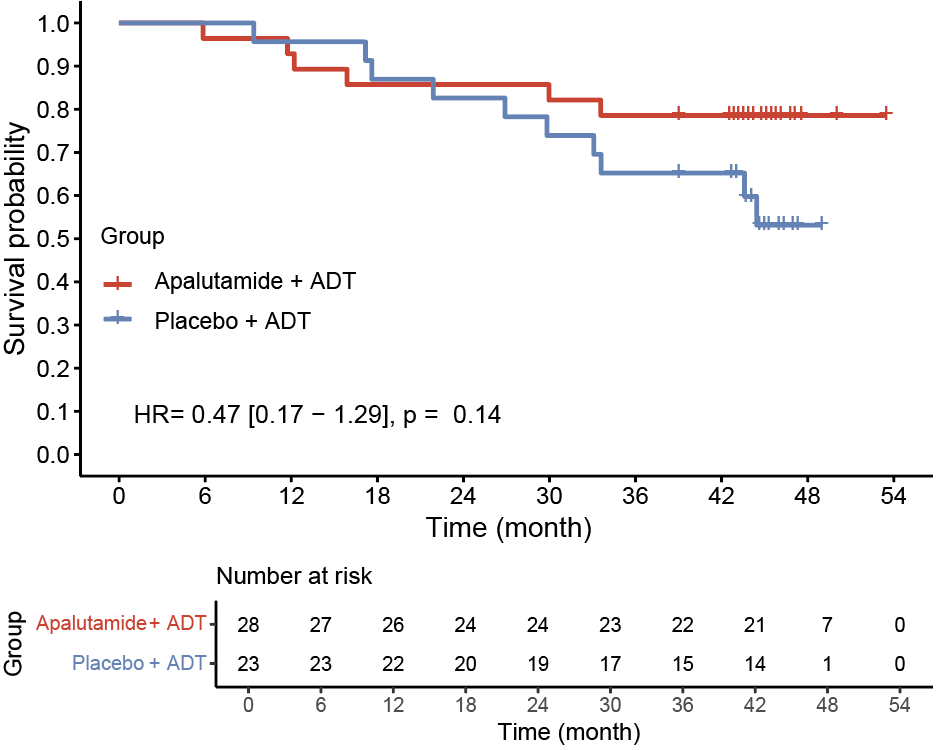


Figure s2. Reconstructed individual patient data (overall survival) in TITAN in Japanese subpopulation reconstructed from Figure 2 in Uemura. Int J Urol. 2022, 29: 533-540. KM, Kaplan-Meier; HR, hazard ratio; CI, confidence interval


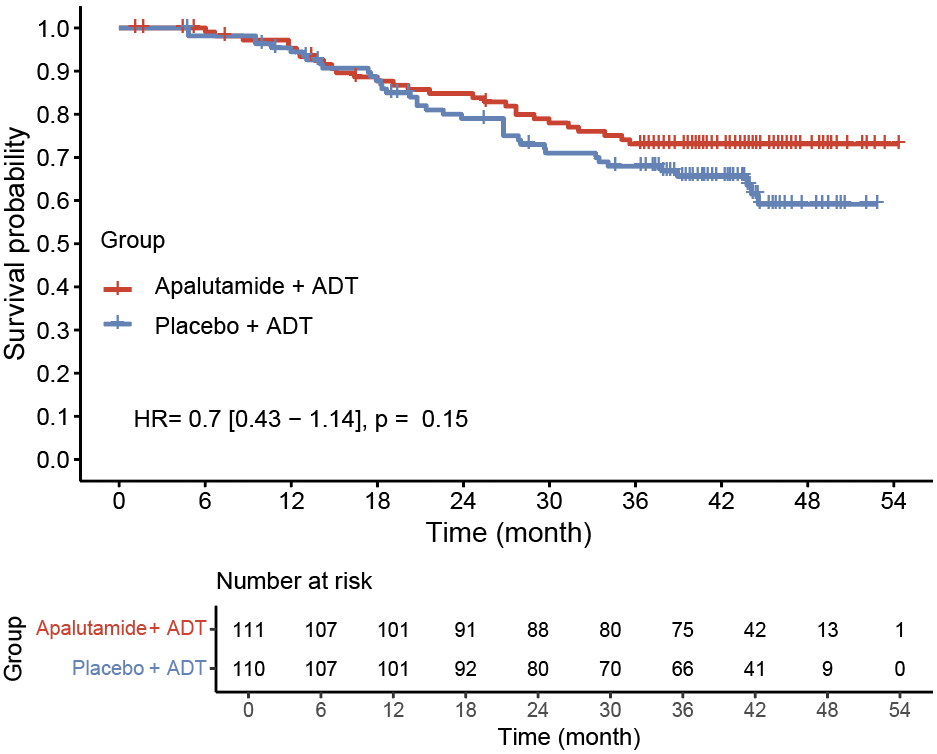


Figure s3. Reconstructed individual patient data (overall survival) in TITAN in Asian subpopulation reconstructed from Figure 2a in Chung. Asian J Androl. 2023, 25: 653-661. KM, Kaplan-Meier; HR, hazard ratio; CI, confidence interval
